# Hepatic and extra-hepatic sequelae, and prevalence of viral hepatitis C infection estimated from routine data in at-risk groups

**DOI:** 10.1186/1471-2334-10-97

**Published:** 2010-04-19

**Authors:** Annunziata Faustini, Paola Colais, Emanuele Fabrizi, Anna Maria Bargagli, Marina Davoli, Domenico Di Lallo, Anteo Di Napoli, Patrizio Pezzotti, Chiara Sorge, Rita Grillo, Carla Maresca, Olga Recchia, Carlo A Perucci, HCV laboratory surveillance Lazio-Region Group

**Affiliations:** 1 Department of Epidemiology, Regional Health System - Lazio Region, Via S. Costanza 53, 00198, Rome, Italy; 2 Public Health Agency, Lazio Region, Italy; 3 Institute of Microbiology, Medicine Faculty, Catholic University, Rome, Italy; 4 Microbiology and Virology Laboratory, S.Filippo Hospital Agency, Rome, Italy; 5 Microbiology and Virology Laboratory, S.Giovanni Hospital Agency, Rome, Italy

## Abstract

**Background:**

Concerns about the hepatitis C virus (HCV) are due to the high risk of chronic liver disease and poor treatment efficacy. Synthesizing evidence from multiple data sources is becoming widely used to estimate HCV-infection prevalence. This paper aims to estimate the prevalence of HCV infection, and the hepatic and extrahepatic sequelae in at-risk groups, using routinely collected data in the Lazio region, Italy.

**Methods:**

HCV laboratory surveillance and dialysis, hospital discharge, and drug-user registers were used as information sources to identify at-risk groups and to estimate HCV prevalence and sequelae.

Full name and birth date were used as linkage keys for the various health registries. Prevalence was estimated as the percentage of cases within the general population and the at-risk groups, with 95% confidence intervals (95% CI) from 1997 to 2001. The risk of sequelae was estimated through a follow-up of hospital discharges up to December 31, 2004 and calculated as the prevalence ratio in HCV-positive and HCV-negative people, within each at-risk group, with 95% CI.

**Results:**

There were 65,127 HCV-infected people in the study period; the prevalence was 1.24% (95%CI = 1.23%-1.25%) in the whole population, higher in males and older adults. Drug users (35.1%; 95%CI = 34.6-35.7) and dialysis patients (21.1%; 95%CI = 20.2%-22.0%) showed the highest values. Medical procedures with little exposure to blood resulted in higher estimates, ranging between 1.3% and 3.4%, which was not conclusively attributable to the surgical procedures. Cirrhosis, hepatocellular carcinoma and encephalopathy were the most frequent hepatic sequelae; cryoglobulinaemia and non-Hodgkin's lymphoma were the most frequent extrahepatic sequelae.

**Conclusions:**

Synthesising data from multiple routine sources improved estimates of HCV prevalence and sequelae in dialysis patients and drug users, although prevalence validity should be assessed in survey and sequelae need a well-defined longitudinal approach.

## Background

Concerns about the impact of the hepatitis C virus (HCV) have arisen from a few very important issues. HCV infection is the most frequent cause of chronic liver disease: up to 85% of infected people have chronic hepatitis [1]; 20% develop hepatic cirrhosis and 1%-4% per year develop hepatocellular carcinoma (HCC) [2]; 20-30% of liver transplants are performed on chronic hepatitis patients [1]; 74-86% of infected people develop persistent viraemia [2], which increases the probability of transmitting the infection. In spite of the recent availability of drugs that completely eliminate the virus in up to 46% of cases [3], mortality associated with HCV infection increases even in industrialized countries [4,5], due to the difficulty in diagnosing first infections and the poor treatment efficacy. No vaccine and no post-exposure prophylaxis are available, therefore studying the risk factors aimed at primary prevention is an essential tool to control HCV infection, as the experience of blood transfusions has shown [6].

After blood screening was introduced, intravenous drug users show the highest prevalence (58% in the US, 79% in the UK) [7,8], followed by dialysis patients (10%-36% in USA and 2%-63% in Europe) [9], transplant patients (9%-33%) [10,11], and patients who underwent surgical procedures [12]. Vertical transmission during childbirth occurs from about 3% to 8% of infected mothers [6,13]. There is no definite evidence of sexual transmission [14,15]. Other exposures reported in the literature, such as, professional exposure [6] and undergoing medical procedures such as endoscopy, intravenous injections and even autologous transfusion [16-18], suggest that infection is possible even with limited bleeding, though associated with very low prevalence or sporadic occurrence.

Adult age at infection, male gender, alcohol consumption and HIV-1 or HBV co-infection are the factors most frequently associated with the chronic sequelae of the disease [2,6]. Viral genotype 1b is twice as frequent in cirrhosis and hepatocellular carcinoma than other genotypes [19]. The role of other factors such as obesity is not consistently supported [20].

Cryoglobulinaemia and non-Hodgkin's lymphoma are the most frequently reported extrahepatic diseases associated with HCV infection; B lymphocyte lymphoma is reported in 5%-8% of cases [21]. Among the more recent extrahepatic disorders diabetes and autoimmune thyroiditis emerged [21,22]. Other extrahepatic disorders have also been reported, but without sufficient confirmation [20,21].

Prevalence is the indicator most frequently used to estimate HCV infection in the population, since the silent onset of HCV infection in most cases makes new infections very difficult to diagnose [1]. Prevalence offers the advantage of estimating the burden of chronic liver disease due to HCV, but the epidemiological characteristics of HCV infection require laboratory testing, which makes estimating prevalence in large populations difficult. Synthesizing evidence from multiple data sources into one model is becoming widely used to estimate hepatitis C prevalence [23].

In this paper, the prevalence of HCV infection and the risk of developing hepatic and extrahepatic sequelae for HCV-infected people within at-risk groups, were estimated synthesising routine data from multiple sources in a region of Central Italy.

## Methods

### Study population

Subjects were residents of Lazio, an Italian region with a population of 5,255,028, which includes the city of Rome. Groups at-risk of HCV infection were defined as dialysis patients, drug users referred to drug treatment services and patients who underwent transfusion, other surgical procedures or transplants between 1 January 1997 and 31 December 2001. Patients who underwent liver transplant and dialysis patients who underwent renal transplant were excluded; those who needed subsequent dialysis were then re-entered in the study as newly exposed people.

Demographic data including age, residence and at-risk conditions came from the health registries.

Details of at-risk group definitions and sources of data for at-risk groups are reported in additional file 1, box 1 and 2, respectively. Patients reported in the regional cause mortality registry (CMR) as having died by 31 December 1996 were excluded.

Human subjects did not participate in the study since only administrative databases were used. On the other hand, our department had been commissioned formally, by the Lazio regional council, to manage the nominative data of infectious disease surveillance, at the time of this study, in accordance also with privacy laws enforced in Italy.

### Estimate of HCV infection prevalence

People were defined as HCV-infected if they were reported to laboratory surveillance as positive to least one enzyme immunoassay (EIA) [7], or were discharged from hospital with a diagnosis of HCV infection, or were reported to the dialysis registry or to the drug-user surveillance as HCV-infected, even if laboratory test results were not available. Additional file 1, box 3, reports details about HCV definition, including ICD-9-CM codes and characteristics of laboratory surveillance and Hospital Discharge Registry (HDR).

Subjects reported in more than one registry and those who underwent more than one invasive procedure were analysed within each individual at-risk group.

Prevalence of HCV infection has been estimated as the percentage of cases in the general population and in each at-risk group with 95% confidence intervals (95%CI), for the period 1997-2001, using the population at the middle of the study period as the denominator. Prevalence was estimated also by gender, age and year using the population at the beginning of each year as denominator. Prevalence was estimated in drug users as a whole group, in injectors and in non- injectors.

Underreporting from laboratories was estimated assuming that laboratories that reported no HCV infection detected a number of HCV infected people equivalent to the mean number reported by their laboratory type. Laboratory compliance by category was: 88% (out of 108) of hospitals, 94% (out of 18) of universities, 72% (out of 43) of outpatient clinics, 39% (out of 148) of private hospitals, 31% (out of 400) of private laboratories. The mean number of HCV-infected people was 420 from hospitals, 104 from universities, 32 from outpatient clinics, 60 from private hospitals and 98 from private laboratories. Subsequently, the expected number of infections were estimated by adding the products of the percentage of non-compliant laboratories and the mean number of infected people in each category to the observed data.

Prevalence trends have been explored only for dialysis patients and drug users. The relative contribution to the prevalence of both new cases and deaths was analysed. A proxy of incidence for these at-risk groups was estimated as the percentage of new HCV diagnoses, by year. Incidence was estimated in these groups thanks to the initial and repeated tests subjects underwent during their frequent diagnostic or therapeutic visits. Mortality rates were estimated in dialysis patients and drug users using data from the regional cause mortality registry.

### Estimates of sequelae

A more specific definition of HCV-positive patients was adopted to estimate sequelae than to estimate prevalence, since the former requires that HCV-infected people have at least one positive laboratory test (immunoassay (EIA), immunoblot test or PCR test for HCV-RNA) reported to the laboratory surveillance system, complete with the test date. Sequelae were studied only in the at-risk groups that showed a higher prevalence of HCV-infection than in the general population. Hepatic [1,2] and extrahepatic [1,2,20,21,24] diseases were analysed as possible sequelae of HCV infection for each at-risk group by individually linking the at-risk group files and the hospital discharge registry (HDR). Diseases were hypothesised to be a consequence of viral infection if it was detected during or before the same hospitalisation at which the sequelae were diagnosed.

The hepatic diseases included cirrhosis, gastrointestinal haemorrhage, haemorrhage due to esophageal varices, liver failure, hepatocellular carcinoma, encephalopathy, portal hypertension. The extrahepatic diseases we considered were cryoglobulinaemia, polyarteritis nodosa, arthritis in psoriasis, pulmonary fibrosis, non-Hodgkin's lymphoma, diabetes, cutaneous porphyria, lichen planus, Sjögren syndrome, Moorhen corneal ulcer, Reynaud's syndrome, Graves' illness, Hashimoto's thyroiditis. Details about ICD-9-CM codes used to define sequelae are reported in the additional file 1, box 3. The sequelae were assessed by means of a follow-up using the discharge diagnoses reported in the hospital files. The follow-up for detecting sequelae ended on December 31^st^, 2004, ranging from 3-8 years per patient.

The relative risk of each sequela was estimated using the prevalence ratio of the specific sequela between HCV-positive and HCV-negative people, and the 95% CI, stratified by at-risk group, from 1997 to 2001.

Initially both the complete name and date of birth were used as linkage keys of the various health registries, and afterwards only the name.

We carried out all the record-linkage between data files using SAS 8.2 software, while STATA 8 was used for the analysis.

## Results

### HCV infection prevalence

There were 65,127 people reported as HCV positive; HCV prevalence in the general population was estimated at 1.24% (95%CI = 1.23-1.25%). It was higher in males (1.45%) than in females (1.04%), and infected males tended to be younger (mean age = 48 yrs) than infected females (mean age = 57 yrs). There was an estimated underreporting of 16,974 (26.1%) HCV-positive people, which increased the estimate of HCV prevalence to 1.56%.

Thirty-six percent of the 65,127 HCV-infected people belonged to a hypothesised at-risk group.

The highest HCV prevalence was found in drug users (35.1%; 95%CI 34.6-35.7) and dialysis patients (21.1%; 95%CI 20.2-22.0) (table 1). Injectors were the most frequent type of drug users (81%) and presented the highest prevalence (42.1%; 95%CI 41.4-42.7). Non- injector drug users showed a slightly higher prevalence than the general population (5.6%; 95%CI 5.0-6.3), suggesting a misclassification of exposure or a life style however linked with a higher risk of HCV infection. The infected drug users were among the youngest exposed subjects (mean age = 33.6 yrs), along with females who underwent obstetric surgery (mean age = 32.1 yrs) and those who underwent an appendicectomy (mean age = 37.9 yrs). Among the patients who underwent invasive medical procedures, those who underwent kidney transplants or blood transfusions or digestive system surgery or gynaecological surgery had a slightly higher prevalence than in the general population but the mean age of infected people showed very high values in these groups. Women who underwent obstetric surgery, including deliveries, and those who underwent an appendicectomy presented a notably lower risk of infection; infected people in both these groups were younger than those who had gynaecological surgery and digestive system surgery, respectively. Age-specific prevalence increased steadily with age for patients who underwent abdominal or gynaecological surgery, but different patterns were observed in at-risk groups with high exposure to blood. Prevalence was higher in 25-74 year-old dialysis patients than in those younger or older, and was higher in 25-54 year-old transfused patients and drug users.

**Table 1:** Characteristics of the study population and prevalence of HCV infection in at-risk groups by age, gender and year, Lazio, 1997- 2001

		drug users			blood	surgery patients				allogenic transplant patients		
	**dialysis**			**not**	**transfus**	**digestive**	**append**	**gynae-**		**lungs**	**bone**	

	**patients**	**all**	**injectors**	**injectors**	**patients**	**system**	**ectomy**	**cological**	**obstetr**	**heart**	**marrow**	**kidney**

**Subjects No.**	8562	29,756	24101	5472	20,841	212,983	32,384	96,480	217,776	127	401	679

% males	60.1	86.4	85.3	90.8	48.3	60.6	46.8			56.7	52.9	61.0

mean age (yrs)	64.0	32.2	32.6	30.5	67.8	57.4	25.0	47.8	31.0	32.6	34.2	39.8

*SD*	*16.3*	*7.9*	7.2	10.4	*18.3*	*17.1*	*16.4*	*14.2*	*5.3*	*21.9*	*17.4*	*14.1*

**HCV infection prevalence**												

No. infected people	1807	10,459	10137	307	1001	7237	270	1251	1342	3	11	52

mean age (yrs)	62.5	34	33.6	34.4	64.7	62.1	37.9	53.7	32.1	42.1	37.5	42.6

*SD*	*15.5*	*7*	*6.6*	*9.1*	*17.0*	*14.9*	*22.0*	*14.1*	*5.6*	*29.9*	*15.0*	*12.0*

prevalence %	*21.1*	*35.1*	*42.1*	5.6	*4.8*	*3.4*	*0.8*	*1.3*	*0.6*	*2.4*	*2.7*	7.7

95% CI	*20.2-22.0*	*34.6-35.7*	*41.4-42.7*	*5.0-6.3*	*4.5 - 5.1*	*3.3 - 3.5*	*0.7 - 0.9*	*1.2 - 1.4*	*0.5 - 0.6*	0.5 - 6.7	*1.4 - 4.9*	5.8 - 9.9

*by age *(yrs)												

<15	13.1	*2.4*	*4.6*	*2.9*	*1.4*	*1.4*	*0.7*	*0.4*	*1.4*	*0*	1.6	*5.0*

15-24	*14.6*	*17.1*	*26.8*	*2.9*	*2.0*	*2.0*	*0.8*	*0.5*	*0.5*	*7.7*	0	*1.5*

25-34	*22.5*	*34.2*	*40.0*	*4.6*	*7.1*	*7.1*	*1.9*	*0.9*	*0.6*	*0*	3.9	*6.5*

35-54	*24.7*	*43.8*	*49.0*	*10.6*	*7.2*	*7.1*	*2.7*	*1.2*	*0.8*	*0*	3.8	*9.0*

55-74	*22.2*	*11.9*	*23.1*	*4.0*	*5.1*	*5.1*	*3.9*	*2.8*	*5.9*	*3.3*	2.3	*9.0*

75+	*17.8*	*0*	*0*	*0*	*3.9*	*3.9*	*4.4*	*3.5*	*0*	*0*	*0*	*0*

*by gender*												

male	*20.9*	*34*	*41.8*	*5.6*	*5.4*	*3.5*	*1.1*			*2.8*	*2.4*	*8*

female	*21.5*	*42.5*§	*47.8*	*6.2*	*4.3*	*3.2*§	*0.56*§	*1.3*	*0.6*	*1.8*	*3.2*	*7.2*

*by year*												

1997	*32.1*	45.8	49.9	*7.7*	*2.3*	*2.9*		*1.2*	0.5	*2.7*	*3.9*	*9.1*

1998	*30.2*	42.3	47.4	*6.4*	*3.3*	*2.7*		*1*	0.4	*0*	*3.1*	*1.4*

1999	*26.5*	39.4	45.3	*6.1*	*2.8*	*2.7*		*1.1*	0.5	*3.8*	*0*	*7.7*

2000	*24.3*	36.8	43.2	*5.5*	*2.4*	*2.5*		*0.9*	0.5	*0*	*0*	*1.9*

2001	*23.1*	34.6	41.6	*5.6*	*3.2*	*2.4*		*0.9*	0.5	*0*	*6.5*	*8.3*

***p-value for trend***	***0.002***	***0.001***	***0.001***	***0.03***	***0.6***	***0.01***		***0.04***	***0.6***			***0.1***

§ statistically significant (p < 0.05)

No gender differences were found for HCV infection prevalence in dialysis patients, or in transfusion or transplant patients. The higher prevalence of HCV infection in female drug users was entirely due to IVDU and may be explained by the greater frequency with which females were tested.

Laboratory surveillance made it possible to detect 314 (17.4%) more HCV cases among dialysis patients than reported in the dialysis register and 3220 (30.8%) more drug users with HCV than reported in the drug-user surveillance, but 700 (38.7%) infections in the former group and 4450 (42.5%) in the latter were not confirmed by available laboratory data. Laboratory surveillance identified an 88.6% HCV prevalence in transfusion patients and up to 90% in those who underwent abdominal (90.9%) or gynaecological surgery (93.8%).

**Figure 1:**
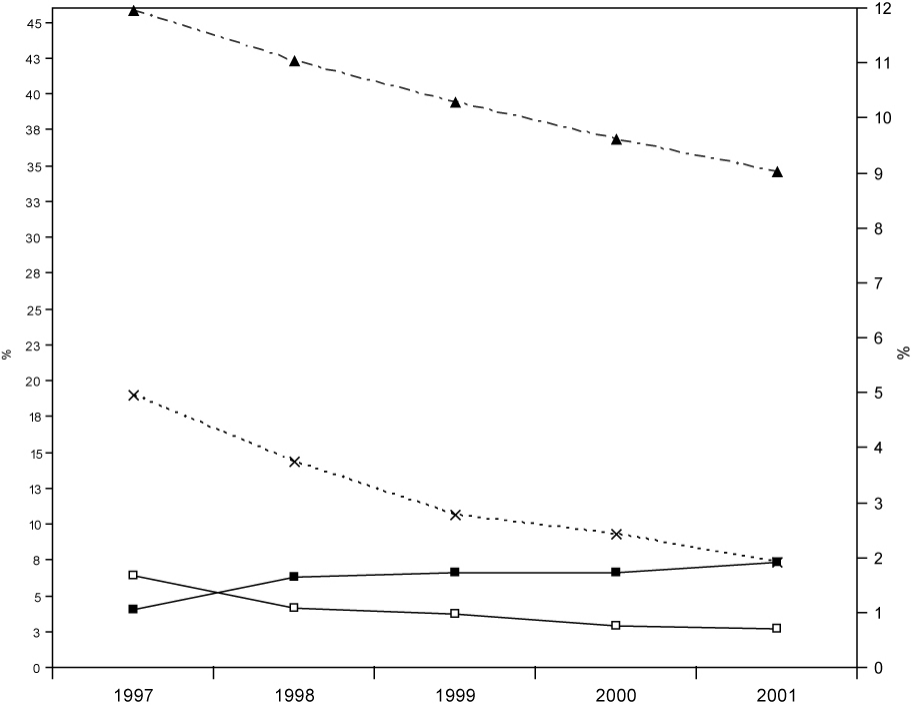
**HCV prevalence, incidence and mortality in drug users, Lazio 1997 - 2001**. Black triangle = HCV prevalence St. Andrew's cross = new HCV diagnoses. White square = mortality in HCV - Black square = mortality in HCV + Y left axis = HCV prevalence (%) and incidence (%) in drug users. Y right axis = mortality (%) in drug users.

**Figure 2:**
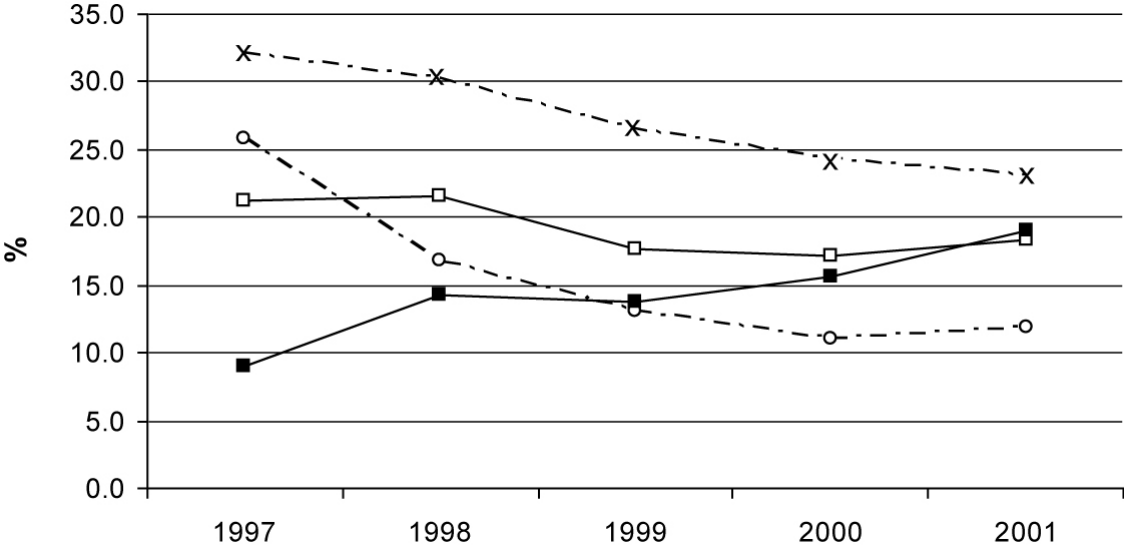
**HCV prevalence, incidence and mortality in patients on dialysis, Lazio 1997-2001**. St. Andrew's cross = HCV prevalence White circle = new HCV diagnoses. White square = mortality in HCV - Black square = mortality in HCV +. Y axis = HCV prevalence, incidence (%) and mortality (%) in patients on dialysis.

The trend of HCV prevalence decreased in drug users (p = 0.001) (figure 1) as well as in dialysis patients (p = 0.002) (figure 2) in the period 1997-2001, with an important reduction of 2.8% and 2.4% in the prevalence rate by year, respectively. The decreasing prevalence in dialysis patients (table 1 and figure 2) might be partly explained by fewer new HCV diagnoses (incidence decreased from 25.9% to 11.9% in the whole period) and, to a greater extent, by the increase in mortality of HCV-infected patients (from 8.9% in 1997 to 19.0% in 2001). A different scenario is suggested for prevalence trends in drug users (table 1 and figure 1). Although mortality increased in the study period for HCV-infected drug users (from 1.1% to 1.9%), the lower number of new cases explains most of the decreasing prevalence (ranging from 18.9% in 1997 to 7.4% in 2001).

The prevalence decreased in patients with digestive surgery (p = 0.01) and gynaecological surgery (p = 0.04) as well (table 1), but with a reduction as small as 0.1 a year. No trend was apparent for transfusion patients, who showed similar prevalence rates over five years. No trend was evident for transplant patients either, who showed a highly variable prevalence in the period under study (table 1). We investigated the spatial and temporal variability of HCV positivity among transplant patients, by hospital and year. For lung/heart or bone marrow transplants we found no differences with respect to the hypothesis that the events were casually distributed. However, most HCV infections in kidney transplant patients were observed in two large hospitals that performed 16 and 18 transplants respectively, 50% of HCV infections from kidney transplants were detected in 1997 and three transplants were performed in the same hospital in the same month of the year.

### Sequelae of HCV infection

The sequelae of HCV infection were studied for 16,671 people (72% of the total 23,432) who had a positive HCV laboratory test and belonged to at least one of the at-risk groups showing a HCV prevalence higher than the general population: dialysis patients, drug users, transfusion patients and those who underwent digestive system surgery or gynaecological surgery. We did not assess sequelae in transplant patients due to the low number of HCV-infected people (table 2).

**Table 2:** Prevalence and prevalence ratios of hepatic and extra-hepatic sequelae in HCV+ and HCV- by at-risk group, Lazio 1997-2001

	patients to dialysis†					drug users §			injector drug users §			
	**HCV +**	**HCV -**	**ratio**	**95% CI**	**HCV +**	**HCV -**	**ratio**	**95% CI**	**HCV +**	**HCV -**	**ratio**	**95% CI**

**N**	**1107**	**7455**			**6009**	**23747**			**5755**	**18346**		

*%*	*100*	*100*			*100*	*100*			*100*	*100*		

**hepatic**	120	451			447	382			427	309		

**diseases**	*10.4*	*6.1*	1.8	1.5 - 2.2	*7.4*	*1.6*	4.6	4.0 - 5.3	7.4	1.7	4.4	3.8 - 5.1

Cirrhosis	48	95			389	266			370	218		

	*4.3*	*1.3*	3.4	2.4 - 4.9	*6.5*	*1.1*	5.8	4.9 - 6.8	6.4	1.2	5.4	4.6 - 6.4

Gastric	73	339			83	87			81	66		

hemorrhage	*0.3*	*4.6*	1.5	1.1 - 1.9	*1.4*	*0.4*	3.8	2.8 - 5.1	1.4	0.4	3.9	2.8 - 5.5

Hemorrhage												

due to eso-	3	13			25	12			13	10		

phageal varix**	*0.3*	*0.2*	1.6	0.3 - 5.7	*0.4*	*0.1*	7.6	3.7 - 16.2	0.2	0.1	4.1	1.7 - 10.6

Liver failure	10	37			43	58			43	46		

	*0.9*	*0.5*	1.8	0.8 - 3.7	*0.7*	*0.2*	2.9	1.9 - 4.4	0.7	0.3	3.0	1.9 - 4.6

Hepato cellular	4	6			17	7			15	6		

carcinoma	*0.4*	*0.1*	4.5	0.9 - 18.9	*0.3*	*0.03*	9.6	3.8 - 27.4	0.3	0.03	8.0	2.9 - 25.1

Hepatic	9	17			105	56			101	46		

encephalopathy	*0.8*	*0.2*	3.6	1.4 - 8.5	*1.7*	*0.2*	7.4	5.3 - 10.4	1.8	0.3	7.0	4.9 - 10.1

Portal	5	6			27	22			24	18		

hypertension	*0.5*	*0.1*	5.6	1.4 - 22.1	*0.4*	*0.1*	4.9	2.7 - 8.9	0.4	0.1	5.3	2.7 - 10.3

**extra-hepatic**	15	58			55	48			51	32		

**diseases**	*1.4*	*0.8*	1.7	0.9 - 3.1	*0.9*	*0.2*	4.5	3.0 - 6.8	*0.9*	*0.2*	5.1	3.2 - 8.2

Cryoglobuli-	6	4			18	8			17	8		

naemia††	*0.6*	*0.1*	10.1	2.4 - 48.7	*0.3*	*0.03*	8.9	3.7 - 23.6	0.3	0.04	6.8	2.8 - 18.1

Non-Hodgkin	2	14			25	19			23	15		

lymphoma	*0.2*	*0.2*	1.0	0.1 - 4.2	*0.4*	*0.1*	5.2	2.8 - 10.0	0.4	0.1	4.9	2.4 - 10.1

									**female reproductive**			

	**transfusion**				**digestive system surgery**				**organs surgery**			

	**HCV +**	**HCV -**	**ratio**	**95% CI**	**HCV +**	**HCV -**	**ratio**	**95% CI**	**HCV +**	**HCV -**	**ratio**	**95% CI**

**N**	**1001**	**19840**			**7237**	**205746**			**1251**	**95293**		

*%*	*100*	*100*			*100*	*100*			*100*	*100*		

**hepatic**	327	3418			2120	14661			65	506		

**diseases**	*32.7*	*17.2*	*1.9*	*1.7 - 2.1*	*29.3*	*7.1*	*4.1*	*3.9 - 4.3*	*5.2*	*0.5*	*9.8*	*7.4 - 12.7*

Cirrhosis	245	996			1912	10031			48	137		

	*24.5*	*5.0*	*4.9*	*4.2 - 5.6*	*26.4*	*4.9*	*5.4*	*5.2 - 5.7*	*3.8*	*0.1*	*26.7*	*18.8 - 37.3*

Gastric	148	2585			358	4199			14	232		

hemorrhage	*14.8*	*13.0*	*1.1*	*1.0 - 1.3*	*5.0*	*2.0*	*2.4*	*2.2 - 2.7*	*1.1*	*0.2*	*4.6*	*2.5 - 7.9*

Hemorrhage												

due to eso-	75	292			236	942			5	16		

phageal varix**	*7.5*	*1.5*	*5.1*	*3.9 - 6.6*	*3.3*	*0.5*	*7.1*	*6.2 - 8.2*	*0.4*	*0.0*	*23.8*	*6.8 - 68.0*

Liver failure	19	178			127	1597			7	137		

	*1.9*	*0.9*	*2.1*	*1.2 - 3.4*	*1.8*	*0.8*	*2.3*	*1.9 - 2.7*	*0.6*	*0.1*	*3.9*	*1.5 - 8.2*

Hepato cellular	40	157			468	1520			*8*	*17*		

carcinoma	*4.0*	*0.8*	*5.1*	*3.5 - 7.2*	*6.5*	*0.7*	*8.8*	*7.9 - 9.7*	0.6	0.0	*35.9*	*13.4 - 87.6*

Hepatic	84	305			535	2374			*6*	*33*		

encephalopathy	*1.5*	*8.4*	*5.5*	*4.2 - 7.0*	*7.4*	*1.2*	*6.4*	*5.8 - 7.0*	0.5	0.0	*13.9*	*4.7 - 33.5*

Portal	*40*	*172*			261	1220			*5*	*15*		

hypertension	*4.0*	*0.9*	*4.6*	*3.2 - 6.5*	*3.6*	*0.6*	*6.1*	*5.3 - 7.0*	0.4	0.0	*25.4*	*7.2 - 73.5*

**extra-hepatic**	28	432			132	1465			24	601		

**diseases**	*2.8*	*2.2*	1.3	0.8 - 1.9	*1.8*	*0.7*	2.6	2.1 - 3.1	*1.9*	*0.6*	3.0	1.9 - 4.6

Cryoglobuli-	9	17			54	87			6	9		

naemia††	*0.9*	*0.1*	10.5	4.1 - 24.9	*0.7*	*0.04*	17.7	12.3 - 25.1	*0.5*	*0.01*	50.8	14.9 - 159

Non-Hodgkin	16	340			42	478			10	65		

lymphoma	*1.6*	*1.7*	0.9	0.5 - 1.5	*0.6*	*0.2*	2.5	1.8 - 3.4	*0.8*	*0.1*	11.7	5.4 - 23.0

HCV+ had laboratory confirmation of the infection; nc = not computable;

† as reported in dialysis or hospital discharge registry;

§as reported in drug users registry or in hospital discharge registry;

**including syndromes due to both cirrhosis and portal hypertension.

Hepatic diseases occurred more frequently in HCV-infected than in HCV-negative people in each of the five groups reported in table 2. The highest prevalence ratios were observed in females who underwent gynaecological surgery (PR = 9.8; 95%CI = 7.4-12.7) and in drug users (PR = 4.6; 95%CI = 4.0-5.3). Cirrhosis, hepatocellular carcinoma, encephalopathy and portal hypertension were the most frequent hepatic sequelae. Among the extrahepatic diseases (table 2), cryoglobulinaemia showed the highest prevalence ratio in each of the five groups, while non-Hodgkin's lymphoma was not associated with HCV infection in dialysis or transfusion patients. No significant associations or consistencies between the at-risk groups were observed for other diseases. Complete data for extrahepatic diseases were reported in the additional file 2. The prevalence ratio of hepatic disease increased with age (results not shown), apart from transfusion patients (table 1); the prevalence ratio of extrahepatic sequelae was not associated with the age of infected people (table 1).

## Discussion

### Prevalence estimates and characteristics of HCV infection in the at-risk groups

The HCV prevalence (1.24%) observed in our population is consistent with the estimates of 1.6% reported in the USA [7], 1.3% reported in European countries [25] and 1.3% reported in blood donors in Italy [26], notwithstanding the possible underestimation discussed below in the limitations section.

That the highest prevalence is observed in drug users, dialysis and transfusion patients who had large or repeated direct exposures to blood, has already been reported in most western countries [3]. The very high prevalence, the highest prevalence in young-adults and similar or even higher prevalence in females confirm that these groups are at high risk of HCV infection and define characteristics of HCV infection in these groups that are different from those seen in the general population. Thus, though prevalence was also higher in patients who have undergone surgical procedures with little exposure to blood, these patients showed steadily increasing prevalence with age and higher prevalence in males suggesting a connection with age instead of with the medical procedures themselves. The lower prevalence observed in patients who had an appendectomy or obstetric surgery supports this hypothesis, since they were younger than those who underwent digestive system surgery or gynaecological surgery, whereas the procedures and the surgery scenarios were essentially comparable. The patterns of age-specific prevalence in dialysis patients, drug users and transfusion patients suggest a possible cohort effect with the most HCV transmission occurring in the last 20-40 years, as reported in the US [27] and in Australia [28].

HCV prevalence estimates in children are based on the limited data reported in the literature. A prevalence of 0.2% was reported in 6-11-year-old children in the US in 1999 [29]. HCV-infected children in this study were more likely to have parents who were drug users than any other direct exposure. One hypothetical interpretation of these results involves the vertical transmission of HCV infection, whose estimate ranges from 3% to 8% [6]. IVDU and transfusion are the most frequent exposures reported in the literature in HCV-positive mothers [13,30], but these estimates usually refer to very young children or those surveyed for HIV infection. The older age of the children (up to 14 years) observed here suggests a possible role of living with HCV-positive parents; testing this hypothesis requires specifically designed studies.

The high HCV prevalence in patients who underwent allogenic bone marrow or kidney transplants, the high risk in the youngest patients, and the prevalence fluctuations from year to year suggest that in our region, although the prevalence rates were much lower than the 11-33% reported in the literature [10,11], HCV infections occurred during the transplant procedures. Kidney transplant patients showed the highest values, and possible hospital clusters were observed for these patients. The high rates of HCV infection among transplant candidates [31], the long duration of dialysis and the high doses of immunosuppressants administered after transplant have been reported as the most frequent risk factors for HCV infection [10]. Unfortunately, having observed possible hospital clusters is not sufficient to assess whether infection occurred during or before the transplant itself. A prospective survey of transplant patients could help resolve this issue. The HCV prevalence of 20-30% reported in the literature [1] in candidates for liver transplant led us to exclude liver transplant as an exposure variable.

### Trends of HCV infection in at-risk groups

That reducing the number of new cases contributes more than mortality does to decreasing HCV prevalence in drug users suggests the positive role of preventive measures. The hypothesis that the harm reduction programme implemented in our region in 1994 reduced the number of new HCV infections as well as of HIV-1 has not been directly evaluated [32] and clashes with the results observed in a study in which the preventive programme reduced HIV-1 prevalence while HCV transmission continued at high levels [33]. Although a more recent study [34] found a reduction in HCV prevalence in people who participated in syringe exchange programs, these results cannot be regarded as supportive of effective protection, since they were not obtained from a community trial study design. In contrast, the increasing mortality in HCV positive dialysis patients contributed more than the decreasing incidence to the prevalence trend suggesting possible gaps in the control measures during dialysis. This hypothesis has already been suggested in our country [31,35]. On the other hand, the absence of a decreasing trend in transfusion patients since 1997 suggests improving transfusion safety measures, even though blood screening began in Italy in 1994.

### Sequelae of HCV infection

The number of subjects whose sequelae were studied is smaller than that of subjects considered infected for the purposes of prevalence estimates: this is because the definition of HCV-infected subjects we chose for estimating this outcome is more specific. The temporal sequence imposed in studying sequelae assured that HCV infection was present before the disease diagnosis, but did not allow to define the timing. Defining a time interval consistent with the latency of all the hypothesized sequelae of HCV infection was impossible because the first infection is so difficult to detect, the latency period to develop hepatic sequelae may take up to 20 years, while the latency for non-Hodgkin's lymphoma has not been defined yet.

Some reasons for confidence in our results are that the exposures hypothesised as at risk showed very similar distributions to those reported in the European prospective cohorts based on hospital patients [36], and the sequelae we found are among the most frequently reported ones in previous related studies [1,21].

A few points require further elucidation. Firstly, although the association between HCV infection and non-Hodgkin's lymphoma has been clearly stated in an Italian multicentre case-control study [37], we observed no risk for non-Hodgkin's lymphoma in two at-risk groups. The shorter survival of dialysis patients and the older age of transfused patients than any other group could explain these results, since both of them shortened the disease latency. The second point involves the role of age at infection. It has been reported that older age at infection means a higher risk of cirrhosis [38]. The role that age at infection plays in developing extrahepatic disease has been studied less than the role it plays in hepatic disease. In our study, the risk of both hepatic and extrahepatic disease did not seem related to the age of infected people, indeed sequelae showed high prevalence ratios in the drug users who are likely to have been infected at very young ages.

Further studies need to better understand the risk profile of these diseases.

The final point concerns the stratification by at-risk group we used to estimate the risk of sequelae associated with HCV infection. This approach, in our opinion, reduces the probability that the association between infection and sequelae could be explained by exposure to blood or by the initial disease causing the invasive procedure, since both HCV-positive and HCV-negative people in each at-risk group shared blood exposure and, in most cases, the initial disease. Furthermore stratification by at-risk group could also help to control confounding due to behavioural or environmental factors if we hypothesise that the life styles of people within the same at-risk group is more homogeneous than in the general population. Unfortunately, we did not have individual information on factors known to be related to hepatic and extrahepatic disease, such as alcohol for cirrhosis or benzene for lymphoproliferative disorders.

### Limitations

Important sensitivity limitations may have affected the prevalence estimates. The resultant underestimation is bipartite: while it was possible to estimate the underreporting from laboratories, which allowed us to increase our prevalence estimate from 1.24% to 1.56%, more important sensitivity limitations derive from the number of infections diagnosed prior to 1997 and those undetected, which were estimated in Europe between 10% and 40% [39]. Moreover, although prevalence makes it possible to estimate the burden of chronic liver disease due to HCV, information about virus genotypes would improve this assessment and incidence is still the best indicator to assess the dynamics of HCV infection and the impact of prevention measures [40].

Other limitations affect this study. The possible misclassification of untested people who were classified as HCV-negative may have resulted in underestimating the relative risk of sequelae in HCV-positive subjects. We did not explore the risk of HCV infection from combined exposures as other authors have [9], or adjust the risk of sequelae by analysing the other factors known to be strong predictors of hepatic sequelae, such as alcohol consumption or HIV-1 and HBV co-infections or environmental exposures [2,6,21].

The most important limitation, however, is intrinsic to the method used; the temporal relationship between events can be accurately understood only by means of a longitudinal prospective study.

## Conclusions

The integrated data source approach improved the estimate of HCV prevalence in at-risk groups; thanks to laboratory data, it provides a more reliable estimate of HCV infection sequelae in at-risk groups, despite longitudinal approximation.

Our study 1) confirms that drug users and dialysis patients have the highest risk of HCV infection; 2) shows higher HCV prevalence in subjects with limited exposure to blood, but the HCV is not conclusively attributable to surgical procedures; 3) shows a decreasing trend of HCV infection between 1997 and 2001 in all at-risk groups, except for patients who had blood transfusions and women who underwent obstetric surgery; 4) strengthens the hypothesis of a high risk of hepatic diseases, cryoglobulinaemia and non-Hodgkin's lymphoma associated with HCV infection.

## List of abbreviations

95% CI: 95% confidence intervals; HCV: hepatitis C virus; HCC: hepatocellular carcinoma; HIV: human immunodeficiency virus; HBV: hepatitis B virus; WHO: World Health Organisation; CMR: cause mortality registry; EIA: enzyme immunoassay; HDR: hospital discharge registry; RR: relative risk; OR: odds ratio; IVDU: intravenous drug users; ICD-9: International Classification of Diseases, 9^th ^revision; DRG: diagnosis-related groups; NHS: National Health Service; NGO: non-governmental organisation; PCR: polymerase chain reaction; RIBA: immunoblot assay.

## Competing interests

The authors declare that they have no competing interests.

## Authors' contributions

AF: 1) made substantial contributions to conception and design; 2) made substantial contributions to analysis and interpretation of data; 2) drafted the manuscript; 3) was involved in critical revision regarding its salient intellectual content. PC: 1) assisted in managing the Hospital Discharge Registry; 2) made substantial contributions to the analyses and interpretation of data, with special attention to record linkage between all file data; 3) was involved in critical revision of the manuscript regarding its significant methodological content. EF: 1) regional coordinator of HCV laboratory surveillance; 2) made substantial contributions to the analyses and interpretation of data, 3) was involved in critical revision of the manuscript. AMB: 1) participated in drug-user surveillance; 2) contributed to data analysis; 3) was involved in critical revision of the manuscript. MD: 1) regional coordinator of drug-user surveillance; 2) contributed to data analysis; 3) was involved in critical revision of the manuscript. DDL: 1) regional coordinator of dialysis registry; 2) contributed to data analysis; 3) was involved in critical revision of the manuscript. ADN: 1) participated in dialysis registry management; 2) contributed to data interpretation; 3) was involved in critical revision of the manuscript. PP: 1) participated in dialysis registry management; 2) contributed to data analysis and interpretation; 3) was involved in critical revision of the manuscript. CS: 1) participated in drug-user surveillance; 2) contributed to data analysis, with special attention to record linkage between files; 3) was involved in critical revision of the manuscript. CAP: 1) participated in designing the surveillance systems and health registries; 2) contributed to the study design;3) was involved in critical revision of the manuscript. RG, CM, OR: 1) contributed to HCV laboratory surveillance, providing support for technical and scientific coordination; 2) made important contributions to data interpretation; 3) were involved in critical revision of the manuscript. All the authors have given their final approval of the version to be published.

## Pre-publication history

The pre-publication history for this paper can be accessed here:

http://www.biomedcentral.com/1471-2334/10/97/prepub

## Supplementary Material

Additional file 1 **Definitions of at-risk group, data sources and HCV sequelae**. This file is organised in three boxes. Box 1 reports the definitions of the groups at risk of HCV infection, on the basis of the information available in the routine data used here. The at-risk groups were: 1) the dialysis patients, 2) the drug users, 3) the patients who underwent digestive system surgery, gynaecological surgery or obstetric, transplants of the heart and/or lungs, of bone marrow or of kidney with the Diagnostic Related Group (DRG) codes or the ICD-9-CM surgery intervention codes used to identify them in the hospital discharge registries; 4) the patients who received a blood transfusion identified with the surgery intervention codes. Box 2 describes the characteristics of the regional dialysis register, the regional drug users surveillance, the hospital discharges registry (HDR) and the information available from these sources that allowed to identify the at-risk groups. Box 3 describes the characteristics of the laboratory surveillance of HCV infection used together with HDR as data sources for HCV infection and the characteristics of the regional Cause Mortality Registry (CMR) used together with the HDR as data sources for the HCV sequelae. The ICD-9-CM codes were reported for both the HCV infection diagnosis and the sequelae. Click here for file

Additional file 2 **Prevalence and prevalence ratios of extra-hepatic sequelae in HCV+ and HCV- in at-risk groups. Lazio 1997-2001**. This file reports an additional table (table 3) where the prevalence ratios of all the studied extra-hepatic sequelae are reported in detail. Click here for file
