# Supplementary material for: Hepatic and extra-hepatic sequelae, and prevalence of viral hepatitis C infection estimated from routine data in at-risk groups
Source: BMC Infect Dis. 2010 Apr 19;10:97. doi: 10.1186/1471-2334-10-97 (PMC2867994; doi:10.1186/1471-2334-10-97)
Supplement: Additional file 1 — Definitions of at-risk group, data sources and HCV sequelae. This file is organised in three boxes. Box 1 reports the definitions of the groups at risk of HCV infection, on the basis of the information available in the routine data used here. The at-risk groups were: 1) the dialysis patients, 2) the drug users, 3) the patients who underwent digestive system surgery, gynaecological surgery or obstetric, transplants of the heart and/or lungs, of bone marrow or of kidney with the Diagnostic Related Group (DRG) codes or the ICD-9-CM surgery intervention codes used to identify them in the hospital discharge registries; 4) the patients who received a blood transfusion identified with the surgery intervention codes. Box 2 describes the characteristics of the regional dialysis register, the regional drug users surveillance, the hospital discharges registry (HDR) and the information available from these sources that allowed to identify the at-risk groups. Box 3 describes the characteristics of the laboratory surveillance of HCV infection used together with HDR as data sources for HCV infection and the characteristics of the regional Cause Mortality Registry (CMR) used together with the HDR as data sources for the HCV sequelae. The ICD-9-CM codes were reported for both the HCV infection diagnosis and the sequelae. [file 1471-2334-10-97-S1.DOC]

**Box 1 The at-risk groups were defined as follows:**

1. Dialysis patients:
   - people reported to the regional dialysis register as having undergone haemodialysis or peritoneal dialysis, in outpatient centres or at home;
   - people who received dialysis in hospital due to a chronic renal failure (ICD-9 codes: 39.95 and 54.98 together with 585).
2. Drug users:
   - people reported to the regional surveillance system as users of heroin, cocaine, cannabis,

hallucinogenic drugs, amphetamines and other stimulants;

- - people hospitalised for addiction or poisoning or excessive use of opioids (ICD-9 codes =

304.0, 304.7, 305.5, 965.0), cocaine (ICD-9 codes = 304.2, 305.6, 968.5), cannabis (ICD-9

codes = 304.3, 305.2, 305.3, 969.6), amphetamines and other stimulants (ICD-9 codes =

304.5, 305.7, 969.7); code 292 was used with any 304.X to include psychoses.

1. Patients who underwent:
   - digestive system surgery (DRGs = 146-162, 164,165 170, 171, 191-202) excluding liver

transplant;

- - gynaecological surgery (DRGs = 353-359, 363-365) or obstetric (DRGs = 370-375);
  - transplants of the heart and/or lungs (ICD-9 codes = 33.50, 33.52, 33.6, 37.5), bone marrow (ICD-9 codes = 41.02, 41.03), kidney (ICD-9 code = 55.69).

1. Patients who received a blood transfusion during hospitalisation (ICD-9 code = 99.03).

**Box 2 Data sources for at-risk groups :**

- - The regional dialysis register has been in operation since 1994 and gathers information about patients who receive dialysis in outpatient clinics or at home from all public and private centres working in the region; data about in-hospital treatment are not included. The number of dialysis patients was 720 per million inhabitants. HCV infections are reported to the registry every three months. HCV prevalence decreased from 25% in 1997 to 17% in 2001; the detection of new cases increased from 9% to 11% in 1997-1999, but got as low as 7% in 2001.
  - The regional drug users surveillance was activated in 1992 and collects individual information on all drug users who attend NHS outpatient treatment services run directly by the NHS and by NGOs. In 2001, there were 27516 reported drug users, estimated as 44.1% of those in the population; up to 82% of them are intra-venous drug users (IVDU). HCV infection was tested in 33% of drug users with a prevalence of 26.3% that increased to 31.5% among IVDU.
  - The hospital discharges registry (HDR) was created in 1996 and reports for each patient the final diagnoses and the diagnostic and therapeutic procedures during hospitalisation in all public and private hospitals in the region.

**Box 3 Data sources for HCV infection and sequelae due to infection:**

- The laboratory surveillance of HCV infection was active in the period 1997 - 2001 and collected the individual data of positive HCV tests (EIA, immunoblotting assays or polymerase chain reaction (PCR)). The laboratories’ compliance was 88 % from hospitals, 94% from universities and 72% from outpatient clinics. It was much lower for private hospitals (39%) and private outpatient laboratories (31%). Between 1997 and 2001, 59977 HCV positive people were reported to the surveillance system. Age information was missing in 38.4% of cases.
- The HDR was a source of information for both:
  - HCV infection diagnosis (ICD-9-CM codes = 070.41; 070.44; 070.51; 070.54);
  - Sequelae of HCV infection

- among hepatic diseases: cirrhosis (ICD-9-CM codes = 571.2, 571.5), gastrointestinal

haemorrhage (ICD-9-CM codes = 578.0, 578.1,578.9), haemorrhage due to esophageal varices (ICD-9-CM codes = 456.0, 571.5 and 456.20, 572.3 and 456.20), liver failure (ICD-9-CM codes = 573.8), hepato-cellular carcinoma (ICD-9-CM codes = 155.0), encephalopathy (ICD-9-CM codes = 572.2), portal hypertension (ICD-9-CM codes = 572.3), liver transplant (ICD-9-CM codes = 50.59)

- among extra-hepatic diseases: cryoglobulinaemia (ICD-9-CM codes = 273.2), polyarteritis nodosa (ICD-9-CM codes = 446.0) arthritis in psoriasis (ICD-9-CM codes = 696.0) pulmonary fibrosis (ICD-9-CM codes = 516.3) non-Hodgkin’s lymphoma (ICD-9-CM codes = 202.8), cutaneous porphyria (ICD-9-CM codes = 277.1), lichen planus (ICD-9-CM codes = 697.0), Sjögren syndrome (ICD-9-CM codes = 710.2), Moorhen corneal ulcer (ICD-9-CM codes = 370.07), Renaud syndrome (ICD-9-CM codes = 443.0), Graves’ illness (ICD-9-CM codes = 242.0), Hashimoto thyroiditis (ICD-9-CM codes = 245.2).

- The regional cause mortality registry (CMR) has been in operation since 1984 and reports information on the deaths of residents regardless of where they die, and includes the cause of death.
